# Cryoablation for atrioventricular nodal re-entrant tachycardia associated with persistent left superior vena cava

**DOI:** 10.1016/j.ipej.2021.08.002

**Published:** 2021-08-13

**Authors:** Hiroyuki Miyazawa, Itsuro Morishima, Yasunori Kanzaki, Yoshihiko Kamiya

**Affiliations:** a Department of Cardiology, Ogaki Municipal Hospital, Ogaki, Japan; b Department of ECG Laboratory, Ogaki Municipal Hospital, Ogaki, Japan

**Keywords:** Atrioventricular nodal re-entrant tachycardia, Cryoablation, Cryo mapping, Persistent left superior vena cava, Slow pathway ablation

## Abstract

Catheter ablation for atrioventricular nodal re-entrant tachycardia (AVNRT) in patients with persistent left superior vena cava (PLSVC) is challenging because of anatomical abnormalities of Koch's triangle associated with the enlarged coronary sinus ostium. We present the Case of successful ablation in a patient with PLSVC using the cryoablation technique. The ablation was successfully performed without damaging the conduction system by virtue of “cryomapping” and “cryoadhesion.” Cryoablation is a safe and efficacious alternative to radiofrequency catheter ablation for the treatment of AVNRT associated with PLSVC.

## Highlights

• Catheter ablation for AVNRT in patients with PLSVC is challenging
• We performed successful ablation in a PLSVC patient with the cryoablation technique
• Ablation was successfully performed without damaging the conduction system

## 1 Introduction

Atrioventricular nodal re-entrant tachycardia (AVNRT), the most common supraventricular tachycardia, can be treated with radiofrequency catheter ablation (RFCA) with a high success rate by delivering energy to the posteroinferior region of Koch's triangle. Persistent left superior vena cava (PLSVC) is a rare venous anomaly οbserved in 0.2% of the general population [1]. Patients with PLSVC have a deviation of the slow pathway (SP) and His bundle associated with a significantly enlarged coronary sinus (CS) ostium, thus making catheter ablation for AVNRT challenging, with an increased risk of atrioventricular block (AVB) [2]. Cryoablation is an alternative to RFCA for AVNRT because of the safety and efficacy related to the reversibility of the cryothermal effects [3]. We present a Case of successful ablation performed in a patient with AVNRT associated with PLSVC using the cryoablation technique.

### 1.1 Case report

**Fig. 1:**
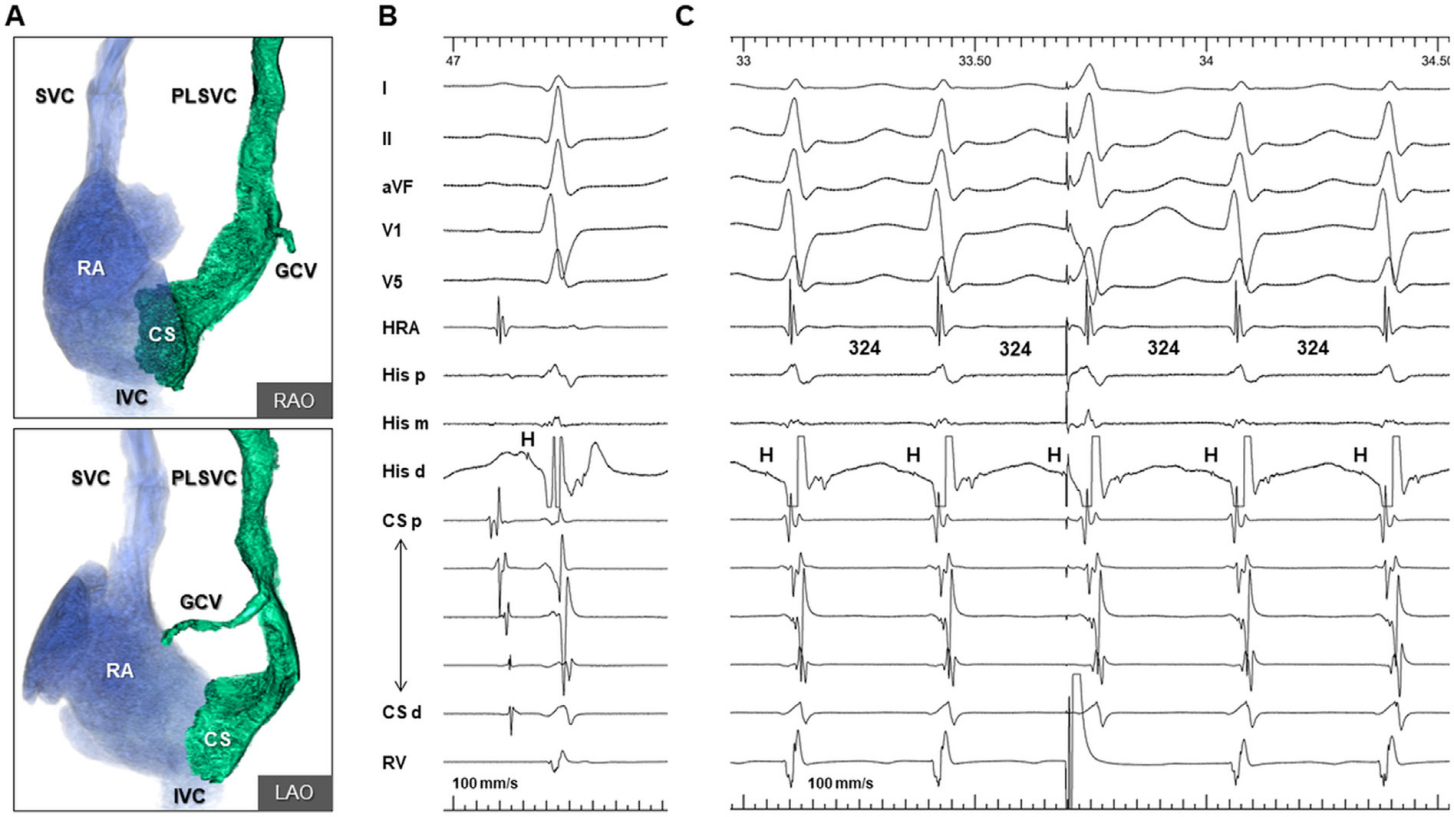
A) Three-dimensional computed tomography in right and left anterior oblique projections showing PLSVC with an enlarged CS ostium. B) Baseline intracardiac electrocardiogram during the sinus rhythm. C) Intracardiac electrogram during the clinical supraventricular tachycardia. The earliest atrial activation was observed at the His catheter with a septal ventriculoatrial time of −4 ms. His-synchronous premature ventricular contraction did not advance the subsequent atrial potentials. RAO, right anterior oblique; LAO, left anterior oblique; SVC, superior vena cava; IVC, inferior vena cava; RA, right atrium; GCV, great cardiac vein; HRA, high right atrium; His, His bundle; RV, right ventricle; H, His electrogram; PLSVC, persistent left superior vena cava; CS, coronary sinus.

**Fig. 2:**
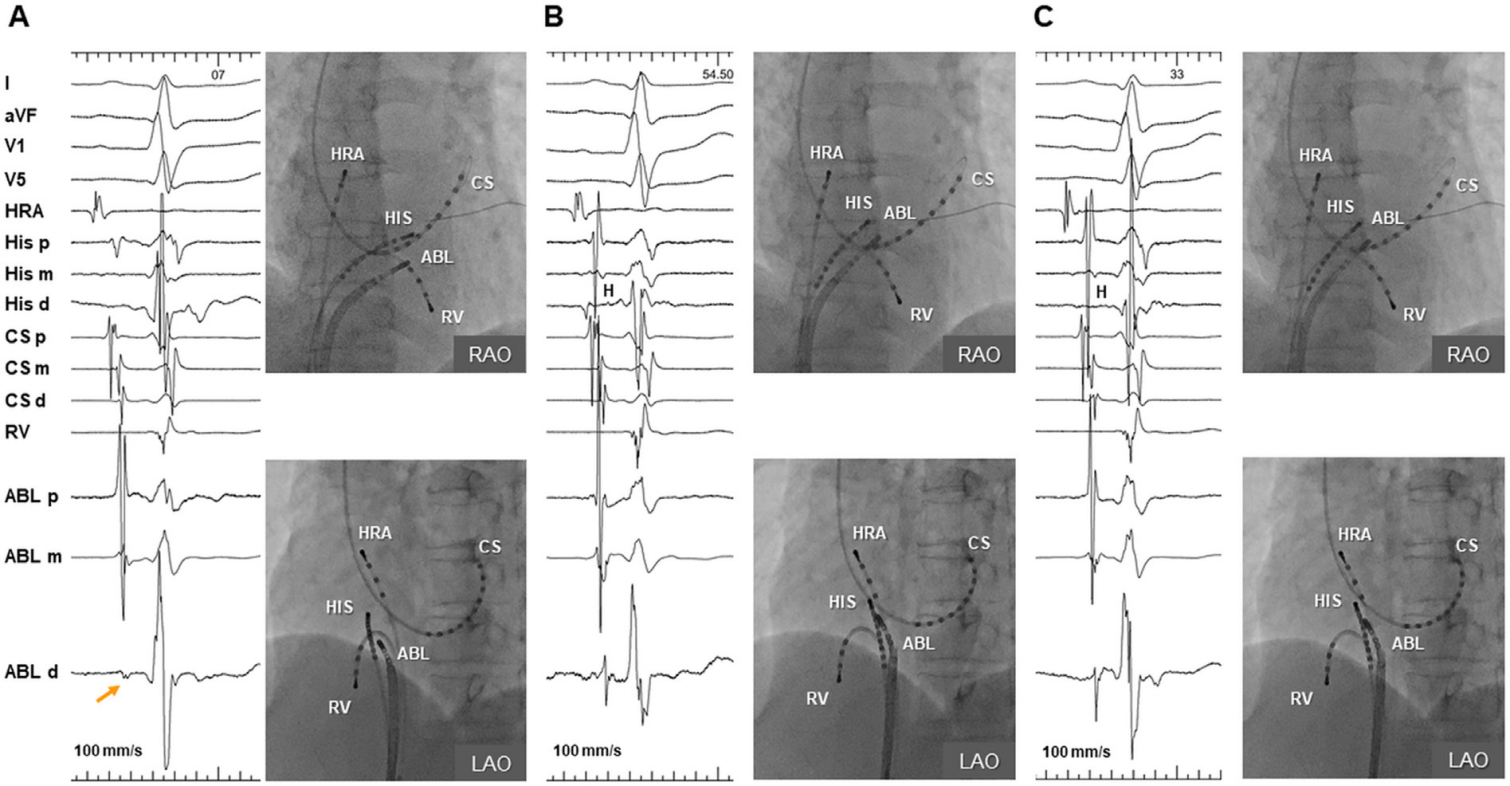
Intracardiac electrocardiograms and fluoroscopic views of cryoablation sites during sinus rhythm. A) The first cryomapping attempt site. The ABL was positioned at the posteroinferior aspect of the CS ostium with an A/V ratio of 0.05. The slow pathway potential (orange arrow) was observed; however, cryomapping at −30 °C for 20 s did not affect the tachycardia. B) The site of successful ablation. The fourth attempt of cryomapping terminated the tachycardia. ABL was positioned at the mid-anterior septum and with an A/V ratio of 0.36. C) The additional cryoablation site. ABL was positioned at the adjacent area to the site of tachycardia termination (B) with an A/V ratio of 0.33. HRA, high right atrium; His, His bundle; RV, right ventricle; RAO, right anterior oblique; LAO, left anterior oblique; H, His electrogram; ABL, ablation catheter; CS, coronary sinus.

**Fig. 3:**
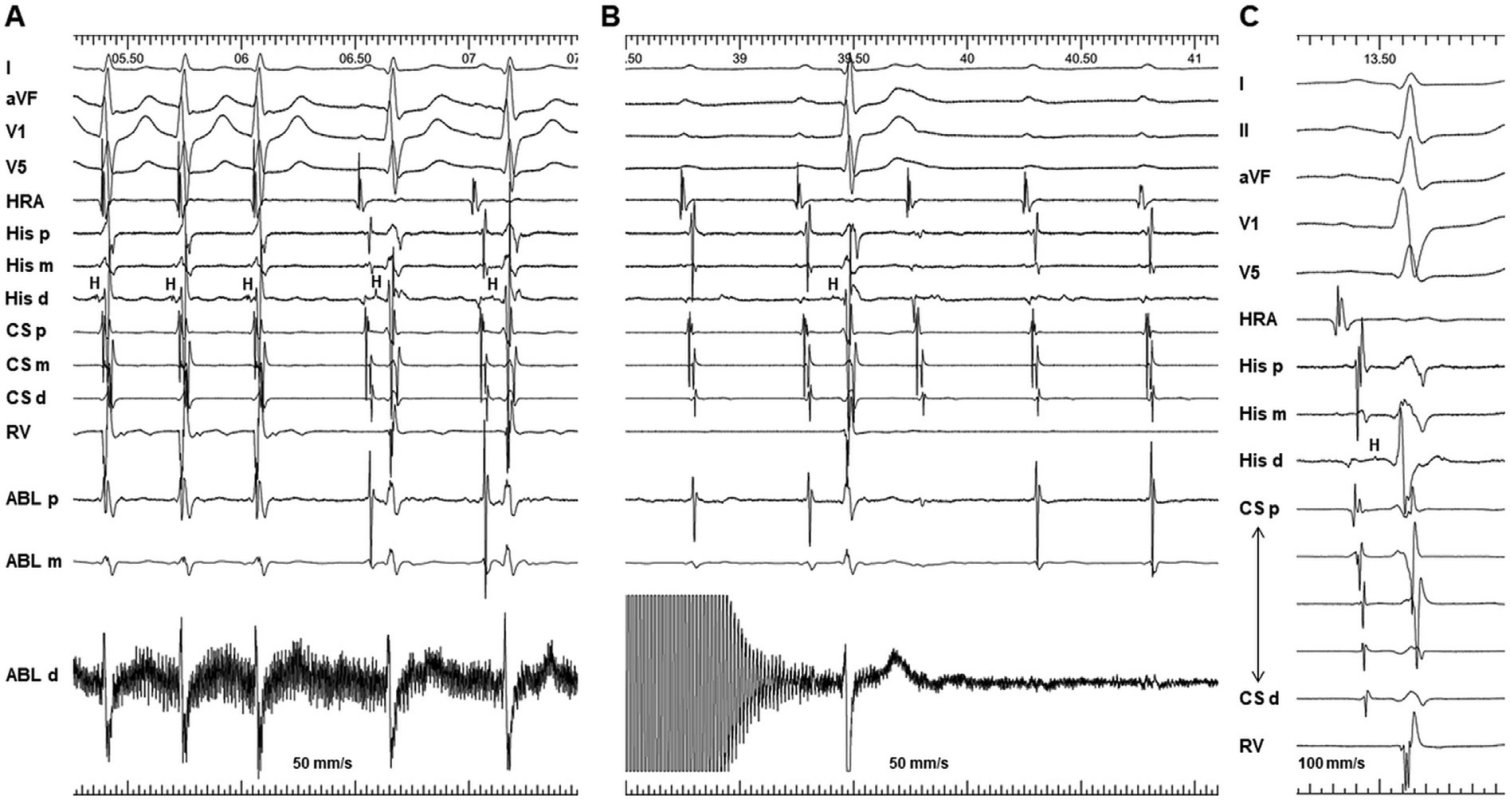
A) The fourth attempt of cryomapping at the mid-anterior septum terminated the tachycardia in 7 s. B) AV block occurred 22 s after cryoablation that was initiated during sinus rhythm. The AV conduction resumed immediately after ablation was discontinued. C) The AH interval after cryoablation remained the same as at baseline. AV, atrioventricular; HRA, high right atrium; His, His bundle; CS, coronary sinus; RV, right ventricle; ABL, ablation catheter; H, His electrogram.

A 62-year-old woman was referred for evaluation and catheter ablation of recurrent paroxysmal narrow-complex tachycardia. Computed tomography revealed coexisting PLSVC with an enlarged CS ostium (29 × 37 mm) (Fig. 1A). After obtaining written consent from the patient, an electrophysiological study was performed in a lightly sedated fasting state without antiarrhythmic drugs. Electrode catheters were positioned in the right atrium, His bundle, and right ventricular apex and dilated CS. At baseline, the patient exhibited normal AH (94 ms) and HV (36 ms) intervals with a sinus cycle length (CL) of 764 ms (Fig. 1B). Atrial programmed stimulation revealed dual AV nodal physiology, and clinical tachycardia (Fig. 1C) was reproducibly induced and terminated by right atrial (RA) pacing. The tachycardia CL was 324 ms, and the earliest atrial activation was observed at the His catheter with a septal ventriculoatrial time of −4 ms. The response to ventricular entrainment pacing exhibited a V-A-H-V pattern. His-synchronous premature ventricular contractions were placed repeatedly without advancing the subsequent atrial potentials. Based on the aforementioned findings, we made a diagnosis of typical “slow-fast” AVNRT. SP ablation was performed using a 6-mm-tipped electrode cryoablation catheter (Freezor Xtra; Medtronic, Minneapolis, MN, USA), considering the catheter stability and safety profile. We initiated cryomapping at the posteroinferior aspect of the CS ostium during tachycardia, where SP potential was observed during the sinus rhythm (Fig. 2A). However, cryomapping at −30 °C for 20 s did not affect the tachycardia. Then, we gradually moved the ablation catheter anteriorly closer to the compact AV node and repeated cryomapping in the same manner. The fourth attempt of cryomapping at the mid-anterior septum (Fig. 2B), where the AV ratio was 0.36 during the sinus rhythm, terminated the tachycardia in 7 s (Fig. 3A). Subsequently, cryoablation with a goal temperature of −80 °C for 240 s was initiated during the sinus rhythm. The AV block occurred after 22 s at the tip temperature of −80 °C (Fig. 3B), and cryoablation was discontinued. The AH conduction returned to normal after 17 s. Cryoablation was applied to the adjacent area with an AV ratio of 0.33 (Fig. 2C) for 240 s during RA pacing, while ensuring a constant AH interval. After a 30-min waiting period, we confirmed the loss of SP conduction, and tachycardia became no longer inducible. The AH interval remained the same as the baseline value (Fig. 3C). During the 12-month follow-up period, the patient remained free of arrhythmia recurrence without receiving medications.

## 2 Discussion

RFCA of AVNRT showed a high success rate by delivering energy to the posteroinferior region of Koch's triangle. However, it still carries an approximately <1% risk of complete AVB, necessitating permanent pacing [4]. The success rate of RFCA for AVNRT associated with PLSVC was reported to be 86%, which was lower than that of the normal heart [5]. To date, several reports are available for catheter ablation of AVNRT associated with PLSVC [6,7]. An enlarged CS ostium due to PLSVC makes SP ablation challenging; the location of the SP and His bundle area may be displaced, increasing the risk of damage to AV nodal conduction during ablation of the SP [2,6]. Moreover, the contact of the ablation catheter with the tissue may have been insufficient because of the difficulty in understanding the anatomical relationship between Koch's triangle and the CS [8].

To overcome these difficulties, we decided to use cryoablation as an alternative to RF ablation [3]. The main advantages of cryoablation are its efficacy and safety regarding reliable SP ablation and inadvertent AV block [9]. The efficacy of cryoablation can be predicted by creating a reversible lesion at a target temperature of −30 °C that helps ascertain whether the target site is appropriate before a permanent lesion is created (cryomapping) [9]. This should minimize the number of unnecessary permanent lesions, and the risk of AVB can be reduced. In the present Case, we could efficiently map the target site from the posterior to mid-anterior septum within the unfamiliar anatomy of the enlarged Koch's triangle because safety was ensured. Complete AVB occurred during cryoablation; however, it was immediately reversed after the termination of freezing. As observed on the electrogram, the AV ratios in cryoablation were higher than those in RFCA, which was also helpful. It was reported that the AV ratio of the successful cryoablation sites was >0.2 in 86% of the sites and was 0.36 in our successful site [3]. Generally, physicians are skeptical to perform ablation high in the septum close to the compact AV node because of the greater AVB risk. However, with cryoablation, such sites can be safely approached. Furthermore, precise contact of the catheter tip at the target site (cryoadhesion) helps in stabilizing the catheter's positioning during the delivery of cryothermal energy [9]. This helps in eliminating the “brushing effects” that occur during the normal beating of the heart and with respiratory fluctuations. This is particularly beneficial in cases where the arrhythmogenic substrate is at a site where contact is difficult to maintain or where nearby tissue ablation is considered dangerous, as it was in our case.

## 3 Conclusion

To the best of our knowledge, this is the first report of a Case of AVNRT in a patient with PLSVC, which was successfully treated with cryoablation. Cryoablation may be a safe and efficacious alternative to RF ablation for such cases.

## Disclosures

None

## Sources of financial support

None.

## Tweet message

Radiofrequency catheter ablation (RFCA) for atrioventricular nodal re-entrant tachycardia (AVNRT) is challenging in patients with persistent left superior vena cava (PLSVC). Here, we describe a Case of AVNRT associated with PLSVC, which was successfully treated with cryoablation. We believe that this is the first case report, to our knowledge, to show that cryoablation can be a safe and efficacious alternative to RFCA for such cases.

## Declaration of competing interest

None.
